# Lower Nr5a2 Level Downregulates the β-Catenin and TCF-4 Expression in Caerulein-Induced Pancreatic Inflammation

**DOI:** 10.3389/fphys.2019.01549

**Published:** 2020-01-09

**Authors:** Ya Mei Sun, Shuai Zheng, Xue Chen, Feng Gao, Jie Zhang

**Affiliations:** ^1^Department of Gastroenterology, Beijing Anzhen Hospital, Capital Medical University, Beijing, China; ^2^Beijing Institute of Heart Lung and Blood Vessel Diseases, Beijing, China

**Keywords:** Nr5a2, AR42J, caerulein, pancreatic inflammation, β-catenin

## Abstract

Nuclear receptor subfamily 5 group A member 2 (Nr5a2) is widely involved in the physiological and pathological processes of the pancreas. However, the cytological and molecular evidence regarding how Nr5a2 implicated in acute pancreatitis (AP) remains insufficient. Here, we explored this problem by using cellular AP model in both normal and Nr5a2 silenced AR42J pancreatic acinar cells. An *in vitro* cellular model of AP was established by stimulating AR42J cells with caerulein (CAE) for 24 h. Reduced Nr5a2 expression was observed in the CAE-treated cells. Nr5a2 silencing led to AP-like inflammation, with increased interleukin (IL)-1β, IL-6, and tumor necrosis factor (TNF)-α mRNA levels. In the cellular AP model, Nr5a2 silencing further increased IL-1β, IL-6, and TNF-α mRNA levels, as well as amylase activity. In addition, we found that Nr5a2 silencing did not affect IL-10 level under physiological conditions but inhibited the anti-inflammatory response of IL-10 in AP model. Moreover, in CAE-induced pancreatic inflammation, Nr5a2 silencing increased the apoptosis and necrosis of acinar cells and inhibited the proliferation of acinar cells, which has not been shown previously. Further experiments showed, for the first time, that Nr5a2 silencing downregulated the expression of β-catenin and its downstream target gene T-cell factor (TCF)-4 in the cellular AP model but increased the expression of nuclear factor (NF)-κB. In conclusion, in CAE-induced pancreatic inflammation, lower Nr5a2 level leads to downregulation of β-catenin and its downstream target gene TCF-4 and upregulation of NF-κB, which exacerbates the inflammatory response and cell damage and inhibits the proliferation and regeneration of acinar cells.

## Introduction

Acute pancreatitis (AP) refers to acute inflammation of the pancreas initiated in pancreatic acinar cells, it is one of the most frequent gastrointestinal causes of hospital admission, with an increased annual incidence of 13–45/100,000 people (Yadav and Lowenfels, 2013). The severity and mortality of this disease vary widely. The mortality rate of mild AP is low, but that of severe AP is >30% due to persistent organ failure and pancreatic necrosis (Banks et al., 2013). Therefore, uncovering the cellular damage-repair mechanisms of the acinar cells involved in mild/severe AP development may have important therapeutic implications.

Nuclear receptor subfamily 5 group A member 2 (Nr5a2), is a member of the orphan family of nuclear receptors and it is highly expressed in embryonic stem cells and the adult liver, intestine, pancreas, and ovary (Lazarus et al., 2012). Nr5a2 regulates especially the development of acinar cells in pancreatic exocrine structures without affecting pancreatic endocrine structures (von Figura et al., 2014; Nissim et al., 2016). Nr5a2 is required for the maintenance of acinar cells identity and re-establishing acinar fate during regeneration (von Figura et al., 2014). A recent global transcriptome analysis shows that Nr5a2 undergoes a marked transcriptional switch, from expressing differentiation-specific to inflammatory genes, in Nr5a2^+/–^ mice (Cobo et al., 2018). Nr5a2 was reported to affect inflammatory response of AP (Flandez et al., 2014). These reports suggest that Nr5a2 is widely involved in the physiological and pathological processes of the pancreas. However, cytological and molecular evidence on whether and how Nr5a2 is involved in the occurrence and development of AP is still insufficient.

The Wnt/β-catenin signaling pathway plays a key role in organogenesis and cell regeneration (Krishnamurthy and Kurzrock, 2018) and is essential for the development of pancreatic acinar cells (Murtaugh et al., 2005). Upon activation of the Wnt/β-catenin signaling pathway, Wnt binds to the frizzled-family receptor, leading to decomplexation of β-catenin and its accumulation in the cytoplasm. Free β-catenin associates with T-cell factor (TCF) in the nucleus to modulate the transcription of target genes (Pai et al., 2017). The interaction between Nr5a2 and β-catenin has been reported in studies of different types of cancer (Fletterick, 2017; Li et al., 2017). However, no studies have addressed this interaction in AP.

To investigate the role of Nr5a2 in AP and to identify potential downstream molecules, we created an *in vitro* model of AP by stimulating AR42J pancreatic acinar cells by caerulein (CAE).

## Materials and Methods

### Cell Culture

The AR42J rat pancreatic acinar cell line (CRL-1492) was purchased from the American Type Culture Collection (ATCC; Manassas, VA, United States). Cells were cultured in F-12K medium (ATCC) containing 20% fetal bovine serum (FBS; Thermo Fisher Scientific, Waltham, MA, United States), 100 μg/mL streptomycin, and 100 U/mL penicillin at 37°C under humidified conditions in 5% CO_2_.

### *In vitro* AP Model

As described previously (Jiang and Wang, 2017; Tang et al., 2017), AR42J cells were starved for 12 h in F-12K medium containing only 2% FBS and then stimulated with 10^–7^ mol/L CAE (Sigma-Aldrich, Merck KGaA, Germany) for 24 h to induce AP. Cells in the control group were treated with phosphate buffered saline (PBS).

### Nr5a2 Silencing by Lentivirus (LV)-Mediated Short Hairpin RNA (shRNA)

Three shRNAs were designed to silence the expression of Nr5a2 (Gene ID: 60349). A lentiviral vector carrying the enhanced green fluorescent protein (GFP) gene was used to transduce shRNAs into AR42J cells. AR42J cells (4 × 10^5^/well) were seeded in a six-well plate and infected with genetically manipulated lentiviruses at a multiplicity of infection of ∼30. At 12 h after infection, the medium was replaced with fresh complete medium under standard conditions. At 48 h after infection, the transduction rate was assessed using a fluorescence microscope. At 96 h after infection, the efficiency of silencing was evaluated at the mRNA and protein levels by quantitative reverse transcription polymerase chain reaction (qRT-PCR) and Western blot analysis, respectively.

The sequences of three shRNAs targeting Nr5a2 (sh1, sh2, and sh3) were: 5′-aaACACAGAAGTCGCATTCAA-3′, 5′-gaGC CTCAAGTTCAAGCGAAA-3′, 5′-tgGGGATGTGCCCTACAA TAA-3′, respectively, and that of the shRNA control was 5′-TTCTCCGAACGTGTCACGT-3′.

### Western Blot Assay

The cells were lysed with radioimmunoprecipitation assay lysis buffer (Thermo Fisher Scientific) containing phenylmethanesulfonylfluoride (Beyotime, Shanghai, China). NE-PER^TM^ Nuclear and Cytoplasmic Extraction Reagents (Thermo Fisher Scientific) were used to extract nuclear proteins from AR42J cells. The Pierce^TM^ BCA Protein Assay Kit (Thermo Fisher Scientific) was used to determine the protein concentration. The following primary antibodies were used for Western blot analysis: anti-Nr5a2 (1:1,000; Abcam, Cambridge, United Kingdom), anti-β-catenin (1:1,000; Abcam), anti-TCF-4 (1:1,000, Abcam), anti-glyceraldehyde 3-phosphate dehydrogenase (GAPDH; 1:1,000; Cell Signaling Technology, Danvers, MA, United States), anti-nuclear factor (NF)-κB P65 (1:1,000; Cell Signaling Technology), anti-lamin B1 (1:1,000; YTHX Biotechnology, Beijing, China), anti-Flag (1:800; Sigma-Aldrich), and/or anti-β-actin (1:1,000; Nakasugi Golden Bridge, Beijing, China) antibodies. Secondary IRDye^R^-conjugated goat anti-rabbit antibody (1:5,000) and goat anti-mouse antibody (1:5,000) were purchased from LI-COR Biosciences (Lincoln, NE, United States). The Odyssey Infrared Imaging System (LI-COR Biosciences) was used to quantify the protein band intensities and calculate the relative concentrations of the target proteins.

### qRT-PCR

TRIzol reagent (Thermo Fisher Scientific) was used to isolate total RNA according to the manufacturer’s instructions. The Revert Aid^TM^ First-Strand cDNA Synthesis Kit (Thermo Fisher Scientific) was used to reverse transcribe RNA into cDNA. The mRNA levels were quantified using the 2^∗^All-in-One^TM^ Q-PCR Mix Kit (GeneCopoeia, Rockville, MD, United States) under the following conditions: 10 min initial denaturation at 95°C, followed by 40 cycles at 95°C for 10 s, 60°C for 20 s, 72°C for 15 s, and 72–95°C in increments of 0.5°C every 6 s for melting curve analysis, and 25°C for 30 s.

The following primer sequences were used for qRT-PCR: Interleukin (IL)-1β, 5′-ATCTCACAGCAGCATCTCGACAAG-3′ and 5′-CACACTAGCAGGTCGTCATCATCC-3′; IL-6, 5′-AGGAGTGGCTAAGGACCAAGACC-3′ and 5′-TGCCGAGTAGACCTCATAGTGACC-3′; Tumor necrosis factor (TNF)-α, 5′-GCATGATCCGAGATGTGGAACTGG-3′ and 5′-CGCCACGAGCAGGAATGAGAAG-3′; IL-10, 5′-GGGCTGCCTTCAGTCAAGTG-3′ and 5′-TGCCTGGGGCATCACTTCTA-3′; Amylase, 5′-AATTGATCTGGGTGGTGAGC-3′ and 5′-CTTATTTGGCGCCATCGATG-3′; Nr5a2, 5′-GTCTCAGGTGATCCAAGCGATGC-3′ and 5′-AGTCTGTCGGAGGCAAGGCTAC-3′; β-catenin, 5′-GTTGCTCCACTCCAGGAATGAAGG-3′ and 5′-GCACCAATGTCCAGTCCGAGATC-3′; TCF-4, 5′-CAGGCTTGCCATCTTCAGTCTACG-3′ and 5′-GCTGCTGGCTTGGAGGAAGAATAG-3′; GAPDH, 5′-AGATGGTGAAGGTCGGTGTGAAC-3′ and 5′-CCTTGACTGTGCCGTTGAACTTG-3′.

### Amylase Activity Assay

The colorimetric Amylase Assay Kit (Abcam) was used to assess α-amylase activity. AR42J cells (1 × 10^6^) were resuspended in 500 μL assay buffer and centrifuged at 14,000 rpm speed for 5 min at 4°C. The supernatant was collected for analyzing α-amylase activity. The absorbance at 405 nm was measured in kinetic mode every 3 min for 60 min at 25°C in the dark.

### Flow Cytometry

The PE Annexin V Apoptosis Detection Kit (BD Biosciences, San Jose, United States) was used to detect apoptosis and necrosis in AR42J cells. AR42J cells (1 × 10^6^) were re-suspended in 1 × binding buffer, and 100 μL of the solution (1 × 10^5^ cells) were mixed with 5 μL phycoerythrin (PE) annexin V and 5 μL 7-aminoactinomycin D (7-AAD), gently vortexed, and incubated at 25°C for 15 min in the dark. Each sample was analyzed by flow cytometry (BD Biosciences) within 1 h.

### 5-Ethynyl-2′-Deoxyuridine (EdU) Cell Proliferation Assay

An EdU cell proliferation kit with Alexa Fluor 488 (Beyotime Biotechnology) was used to detect the proliferation of AR42J cells in AP. AR42J cells were treated with 10 μM/mL EdU, after which they were collected, fixed in 4% paraformaldehyde, and incubated with 0.3% Triton X-100. After addition of 0.5 mL click reaction solution (click reaction buffer, CuSO_4_, Azide488, click additive solution), the cells were incubated for 30 min at room temperature in the dark. The samples were subjected to flow cytometry (BD Biosciences), and fluorescence was measured using 488 nm lasers and analyzed by Flowjo software.

### Statistical Analysis

Data are shown as means ± SEM. Differences between groups were assessed by *t*-test, and differences among groups were examined by one-way analysis of variance followed by Bonferroni correction. *P* values < 0.05 were taken to indicate statistical significance.

## Results

### Reduced Expression of Nr5a2 Was Observed in an *in vitro* AP Model

**FIGURE 1:**
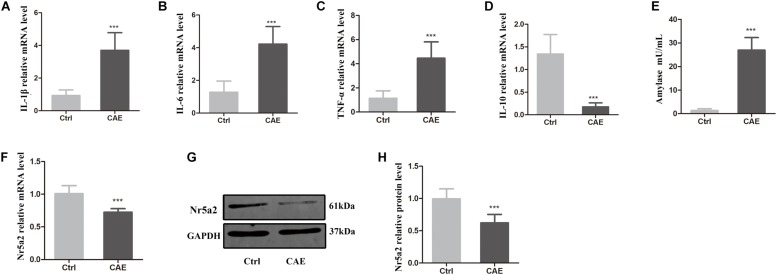
Reduced expression of Nr5a2 was observed in an *in vitro* AP model established by stimulating AR42J cells with caerulein (CAE) for 24 h. **(A–E)** The effects of CAE on the mRNA levels of IL-1β, IL-6, TNF-α, and IL-10 were measured by qRT-PCR, and amylase activity was measured by a colorimetric assay. **(F–H)** The mRNA and protein levels of Nr5a2 in AR42J cells after treatment with CAE were quantified by qRT-PCR and Western blot analysis. Data are shown as mean ± SEM, *n* ≥ 3 in each test, ^∗∗∗^*p* < 0.001 vs. control.

To explore the role of Nr5a2 expression in AP, an *in vitro* model of AP was established. AR42J pancreatic acinar cells were stimulated with CAE for 24 h to induce AP. Then, the pro-inflammatory and anti-inflammatory responses of the cellular AP model were assessed by qRT-PCR, and the amylase activity in AP model was assessed by a colorimetric assay. The average mRNA levels of IL-1β, IL-6, TNF-α in the CAE-treated cells were significantly higher than in the control cells treated with PBS (Figures 1A–C); conversely, the average mRNA level of IL-10 was significantly lower in CAE-treated cells than in the control cells (Figure 1D). As expected, amylase activity was significantly higher in the CAE-treated cells than in the control cells (26.93 ± 4.95 vs. 1.36 ± 0.62) (Figure 1E). These data suggest that the AP model was successfully established *in vitro* by stimulation of AR42J cells with CAE.

To determine how Nr5a2 is involved in AP, the mRNA and protein levels of Nr5a2 were quantified in the AP cell model. qRT-PCR and Western blot analyses showed that the average mRNA and protein levels of Nr5a2 in CAE-treated cells were significantly decreased compared with control cells (Figures 1F–H). It is implied that Nr5a2 expression plays an important role in AP.

### Nr5a2 Silencing Leads to AP-Like Inflammation in AR42J Cells

**FIGURE 2:**
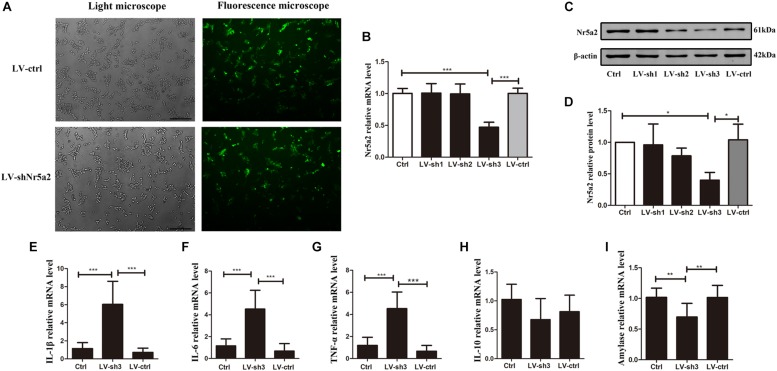
Nr5a2 silencing led to AP-like inflammation in AR42J cells. **(A)** The infection rate of control lentiviral vector or vector carrying shRNA targeting Nr5a2 in AR42J cells was assessed using a fluorescence microscope at 48 h. **(B–D)** The mRNA and protein levels of Nr5a2 were measured at 96 h after infection with the lentivirus. **(E–I)** The effects of Nr5a2 silencing on the mRNA levels of IL-1β, IL-6, TNF-α, and IL-10, and amylase were measured by qRT-PCR. Data are shown as mean ± SEM, *n* ≥ 3 in each test, ^∗^*p* < 0.05, ^∗^*^∗^p* < 0.01, ^∗∗∗^*p* < 0.001.

In order to verify the effect of reduced Nr5a2 level on pancreatic acinar cells, lentivirus-mediated shRNAs were designed to silence the expression of Nr5a2. Up to 80% AR42J cells were infected successfully by lentiviral vector carrying a shRNA targeting Nr5a2 (Figure 2A). qRT-PCR and Western blot data showed that the Nr5a2 mRNA and protein levels of cells treated with lentivirus-mediated sh3 (LV-sh3) were 0.47 ± 0.07 and 0.40 ± 0.10, respectively, which displayed more than 50% decreases compared with controls; meanwhile, the lentiviral vector infection itself did not affect the expression of Nr5a2 (Figures 2B–D). Thus, LV-sh3 was selected to silence Nr5a2 expression in the following experiments.

Interestingly, the downregulated Nr5a2 expression led to AP-like inflammation in AR42J cells. The IL-1β, IL-6, TNF-α mRNA levels were significantly higher in the LV-sh3-infected cells than in the control cells (Figures 2E–G). In contrast to CAE treatment, the decrease in Nr5a2 expression did not significantly alter the level of IL-10 mRNA under physiological conditions (Figure 2H). In addition, the amylase mRNA level was lower in LV-sh3-treated cells than in the control cells (Figure 2I). These parameters indicate that Nr5a2 silencing leads to AP-like inflammation in AR42J cells under physiological conditions, which is mediated by increasing the levels of pro-inflammatory factors rather than reducing anti-inflammatory factor.

### Nr5a2 Silencing Exacerbates the Inflammatory Response and Damage, and Inhibits the Proliferation, of Acinar Cells in the AP Model

**FIGURE 3:**
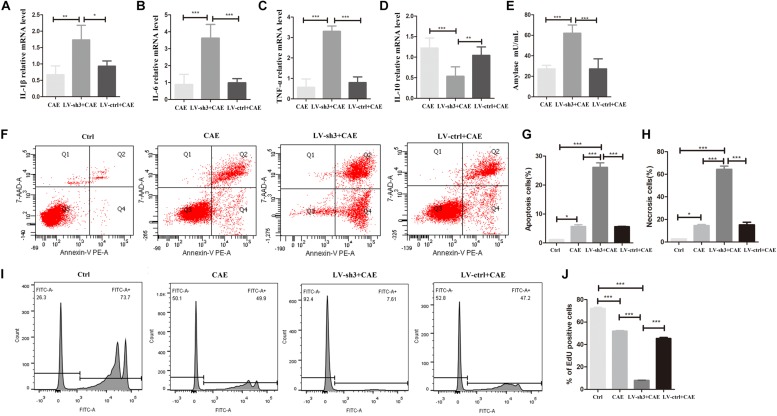
Nr5a2 silencing exacerbated the inflammatory response and damage of acinar cells and inhibited the proliferation of acinar cells in CAE-induced inflammation. **(A–E)** The levels of IL-1β, IL-6, TNF-α, and IL-10, and amylase activity in the AP model are shown. **(F–H)** Apoptosis and necrosis cells were measured by flow cytometry, with living cells presented in the left-lower quadrant (Q3), apoptotic cells in the right-lower quadrant (Q4), necrotic cells in the right-upper quadrant (Q2), and cell debris in the left-upper quadrant of the figure (Q1). **(I,J)** The proliferation of AR42J cells labeled with EdU was measured by flow cytometry. CAE group: control cells treated with CAE for 24 h, LV-sh3 + CAE group: LV-sh3-infected cells treated with CAE for 24 h, LV-ctrl + CAE group: LV-control shRNA-infected cells treated with CAE for 24 h. Data are shown as mean ± SEM, *n* ≥ 3 in each test, *^∗^p* < 0.05, ^∗^*^∗^p* < 0.01, ^∗∗^*^∗^p* < 0.001.

To further clarify the effect of Nr5a2 expression on inflammatory response, damage, and regeneration of acinar cells in the AP model, we examined the expression of inflammatory factors, amylase activity, apoptosis, necrosis, and proliferation of acinar cells in the AP model after silencing Nr5a2. Stimulating LV-sh3-infected AR42J cells with CAE significantly increased the levels of IL-1β, IL-6, and TNF-α (Figures 3A–C) and decreased the level of IL-10 (Figure 3D) compared with the levels in cells treated only with CAE. The amylase level was also significantly increased after CAE treatment in the LV-sh3-infected cells compared with non-infected cells (62.06 ± 7.29 vs. 27.22 ± 2.99) (Figure 3E).

In addition, flow cytometric analysis of LV-sh3-infected cells treated with CAE showed that downregulation of Nr5a2 level markedly exacerbated the damage to acinar cells (Figure 3F). The percentages of apoptotic and necrotic cells were significantly increased in LV-sh3-infected cells treated with CAE (26.13 ± 2.17 and 64.43 ± 3.92%, respectively) compared with the CAE only treated cells (5.70 ± 0.85 and 14.57 ± 1.39%, respectively) (Figures 3G,H).

Moreover, the flow cytometric results showed that the proportion of EdU-positive cells was significantly reduced in Nr5a2 silenced cells treated with CAE (7.97 ± 0.36%) compared with cells treated with CAE only (51.48 ± 1.25%), and even the cells were almost at a standstill (Figures 3I,J), which indicated that the proliferation of acinar cells in AP model was markedly inhibited after silencing Nr5a2.

These results demonstrate that lower Nr5a2 level in acinar cells further exacerbates inflammatory response and damage, and inhibits the proliferation, of acinar cells in the AP model.

### Effects of Nr5a2 Downregulation on β-Catenin, TCF-4, and NF-κB Expression in AP

**FIGURE 4:**
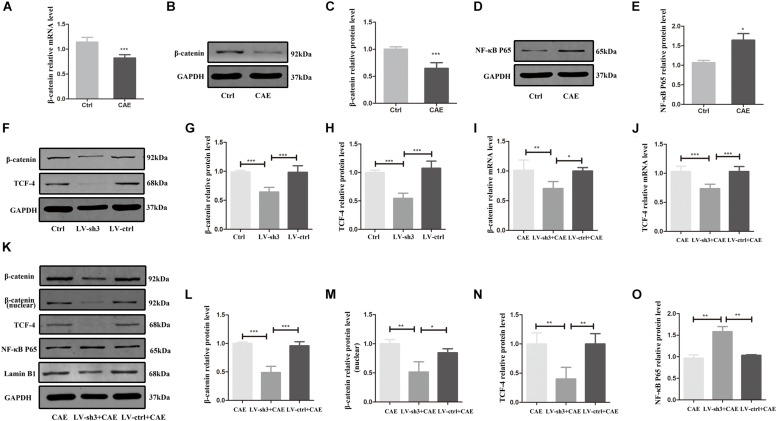
The expression of signaling molecules downstream of Nr5a2. **(A–E)** In the CAE-treated cells, the mRNA and protein levels of β-catenin and the protein level of NF-κB P65 were measured. **(F–H)** In Nr5a2 silenced AR42J cells, the protein levels of β-catenin and TCF-4 were measured. **(I–O)** In cellular AP model after silencing Nr5a2, the mRNA and total/nuclear protein levels of β-catenin, the mRNA and protein levels of TCF-4, the protein level of NF-κB P65 are shown. CAE group: control cells treated with CAE for 24 h, LV-sh3 + CAE group: LV-sh3-infected cells treated with CAE for 24 h, LV-ctrl + CAE group: LV-control shRNA-infected cells treated with CAE for 24 h. Data are shown as mean ± SEM, *n* ≥ 3 in each test, ^∗^*p* < 0.05, ^∗^*^∗^p* < 0.01, ^∗∗^*^∗^p* < 0.001.

To determine how Nr5a2 downregulation exacerbates inflammation in AP, we evaluated the expression of signaling molecules downstream of Nr5a2. In cells treated with CAE, the mRNA and protein levels of β-catenin were significantly lower (Figures 4A–C), and that of NF-κB P65 higher compared with control cells (Figures 4D,E). Furthermore, in Nr5a2 silenced cells, we also observed downregulation of β-catenin and TCF-4 at the protein level (Figures 4F–H).

To further confirm that β-catenin and NF-κB are affected by Nr5a2 expression in AP, we also evaluated the expression of β-catenin and downstream molecule TCF-4, and NF-κB P65 in Nr5a2 silenced cellular AP model. The mRNA and protein levels of β-catenin and TCF-4 were significantly lower in the cells treated with LV-sh3 and CAE than in cells treated with CAE only (Figures 4I–L,N). And the protein level of nuclear β-catenin was also decreased in the LV-sh3-infected cells treated with CAE (Figures 4K,M). In addition, Western blot analysis showed that the protein level of NF-κB P65 was increased in the LV-sh3 and CAE treated cells compared with CAE only treated cells (Figure 4O). These results support that lower Nr5a2 level leads to downregulation of β-catenin and its downstream target gene TCF-4 and to upregulation of NF-κB, which exacerbates inflammatory response and cell damage and inhibits the proliferation and regeneration of acinar cells in CAE-induced pancreatic inflammation.

## Discussion

Nr5a2 has been revealed that it could regulate the development of, and homeostasis in, the pancreas and maintain the differentiation and exocrine function of acinar cells (von Figura et al., 2014). In a recent study, mice with loss of one Nr5a2 allele in their pancreatic acinar cells exhibited a more severe pancreatic inflammatory response and increased levels of pro-inflammatory cytokines (Flandez et al., 2014). Further exploration and study of Nr5a2 in AP and the related molecular mechanisms is of great significance for therapy of AP.

By stimulating AR42J cells with CAE, we successfully mimicked the pathogenesis of AP *in vitro*. The stimulated AR42J cells showed increased levels of pro-inflammatory factors and amylase and decreased level of anti-inflammatory factor. As an *in vitro* model, AR42J cell line has been widely used to investigate the secretion, proliferation, and apoptosis of pancreatic acinar cells (Masamune et al., 2001), as it maintains the characteristics, parallel receptor expression, and signal transduction mechanisms of normal pancreatic acinar cells (Christophe, 1994). During the onset of AP, inflammatory factors originating from damaged pancreatic acinar cells are released to recruit leukocytes (Gu et al., 2013; Habtezion, 2015). Our results are in accordance with these reports, suggesting that Nr5a2 roles in regulating inflammation.

A recent transcriptome analysis study reported that 91% of genes expressed in the pancreas were upregulated in Nr5a2^+/–^ mice, and that 68% of these genes were associated with inflammatory pathways. In addition, Nr5a2 haploinsufficiency mimicked AP at the transcriptome level (Cobo et al., 2018). In our *in vitro* AP model, the expression of Nr5a2 was reduced, and Nr5a2 silencing led to AP-like inflammation in acinar cells, with increased levels of pro-inflammatory factors and decreased level of amylase mRNA, which was consistent with the above study. However, we also found that Nr5a2 silencing did not affect IL-10 level under physiological conditions but did inhibit the anti-inflammatory response of IL-10 in AP, which has not been proposed.

Moreover, we also evaluated the effect of lower Nr5a2 level on apoptosis, necrosis and proliferation of acinar cells in the AP model. The regeneration of acinar cells following AP was assessed by measuring the proliferation of acinar cells. Pancreatic acinar cells can regenerate damaged tissue by initiating a proliferative process after AP (Bombardo et al., 2018). Studies suggest that new acinar cells are regenerated almost exclusively from the division and self-duplication of pre-existing acinar cells (Desai et al., 2007; Houbracken and Bouwens, 2017). The proliferation was used to assess regeneration of acute pancreatitis (Keefe et al., 2012). The ability of acinar cells to regenerate and proliferate is an important factor in determining the progression and prognosis of AP (Chan et al., 2017). And, it is first proved that Nr5a2 silencing affected not only inflammatory factors but also apoptosis, necrosis, and proliferation of acinar cells in AP, which promoted the progression of AP.

Among the molecules downstream of Nr5a2 signaling in AP, we found decreased β-catenin and increased NF-κB P65 levels in the AP model. These results suggest that a potential mechanism of Nr5a2 regulation of inflammation in acinar cells is its interaction with β-catenin and NF-κB. Further results showed that β-catenin and TCF-4 levels were significantly reduced in the Nr5a2 silenced AP model. Since β-catenin has been reported to promote the maintenance and proliferation of mature acinar cells under normal and AP conditions (Keefe et al., 2012), and reduce the severity of AP by decreasing pancreatic acinar cells necrosis in, and inflammatory factors release from, pancreatic acinar cells (Huang et al., 2019), while NF-κB is known to play key roles in the pathogenesis and progression of AP (Rakonczay et al., 2008; Jakkampudi et al., 2016), and activation of NF-κB in the pancreas can induce an inflammatory response in pancreatic acinar cells (Chen et al., 2002). Therefore, our results suggest lower Nr5a2 level regulates pancreatic inflammation via β-catenin and NF-κB expression.

We attempted to overexpress Nr5a2 using two kinds of lentivirus. We successfully achieved the overexpression of Nr5a2 at the mRNA level in AR42J cells; however, we failed to detect the expression of Flag at the protein level. Therefore, we chose the method of silencing Nr5a2 to further confirm the role of Nr5a2 in AP. As shown in the Supplementary Figures S1, S2, up to 80% of AR42J cells were infected successfully by the lentiviral vectors, and the Nr5a2 mRNA level was 2.32-fold higher in overexpressed lentivirus-infected cells than in control cells. Furthermore, stimulating the overexpressed lentivirus-infected AR42J cells with CAE decreased the levels of IL-1β, IL-6, and TNF-α. However, we failed to detect the expression of Flag at the protein level. Based on the above results, we cannot conclude that we have successfully achieved the overexpression of Nr5a2 at the protein level in AR42J cells. Since too many factors are involved in the process from transcription to translation, the reasons for the failure of Nr5a2 overexpression at the protein level in AR42J cells require further exploration. We hope to find a solution and supplement these data in future studies.

## Conclusion

During the early stages of AP, pancreatic acinar cells exhibited reduced Nr5a2 expression and the release of pro-inflammatory factors. As AP progressed, the reduced Nr5a2 expression exacerbated apoptosis and necrosis in the acinar cells, resulting in a second wave of blows to the pancreas. Further exacerbation of the AP process inhibited the expression of Nr5a2 and downregulated the expression of β-catenin and TCF-4, which prevented regeneration of acinar cells and maintained more severe pancreatic inflammation. Thus, Nr5a2 is a key molecule involved in the occurrence, development, and outcome of AP. AP therapy may benefit from further studies focused on Nr5a2.

## Data Availability Statement

The datasets generated for this study are available on request to the corresponding author.

## Author Contributions

JZ and FG designed the experiments. YS performed the experiments and wrote the manuscript. XC analyzed the data. SZ and FG revised the manuscript.

## Conflict of Interest

The authors declare that the research was conducted in the absence of any commercial or financial relationships that could be construed as a potential conflict of interest.

## Supplementary Material

FIGURE S1 Construction of the lentiviral vector for Nr5a2 overexpression. **(A)** The component order in the lentiviral vector for Nr5a2 overexpression. **(B)** The infection rate of control lentiviral vector or the Nr5a2-overexpression vector in AR42J cells was assessed using a fluorescence microscope at 48 h. **(C)** Nr5a2 mRNA level after infection with lentiviral vector was measured by qRT-PCR at 5 days. **(D–F)** The effects of Nr5a2 overexpression at the transcriptional level on IL-1β, IL-6, and TNF-α mRNA levels were measured by qRT-PCR. **(G)** Flag protein expression was determined by Western blot after infection with lentiviral vector for 6 days. Positive control: positive control expressing the flag protein, LV-ctrl: infection with the control lentiviral vector, OE: infection with lentiviral vector overexpressing Nr5a2. Data are shown as mean ± SEM, *n* > 3 in each test, ^∗^*p* < 0.05, ^∗∗∗^*p* < 0.001.

FIGURE S2 Re-construction of the lentiviral vector for Nr5a2 overexpression. **(A)** The component order in the lentiviral vector for Nr5a2 overexpression. **(B)** The infection rate of control lentiviral vector or the Nr5a2-overexpression vector in AR42J cells was assessed using a fluorescence microscope at 48 h. **(C)** Flag protein expression was determined by Western blot after infection with the lentiviral vector for 6 days. Data are shown as mean ± SEM, *n* > 3 in each test.
