# Supplementary figures and images for: Lower Nr5a2 Level Downregulates the β-Catenin and TCF-4 Expression in Caerulein-Induced Pancreatic Inflammation
Source: Front Physiol. 2020 Jan 9;10:1549. doi: 10.3389/fphys.2019.01549 (PMC6962314; doi:10.3389/fphys.2019.01549)

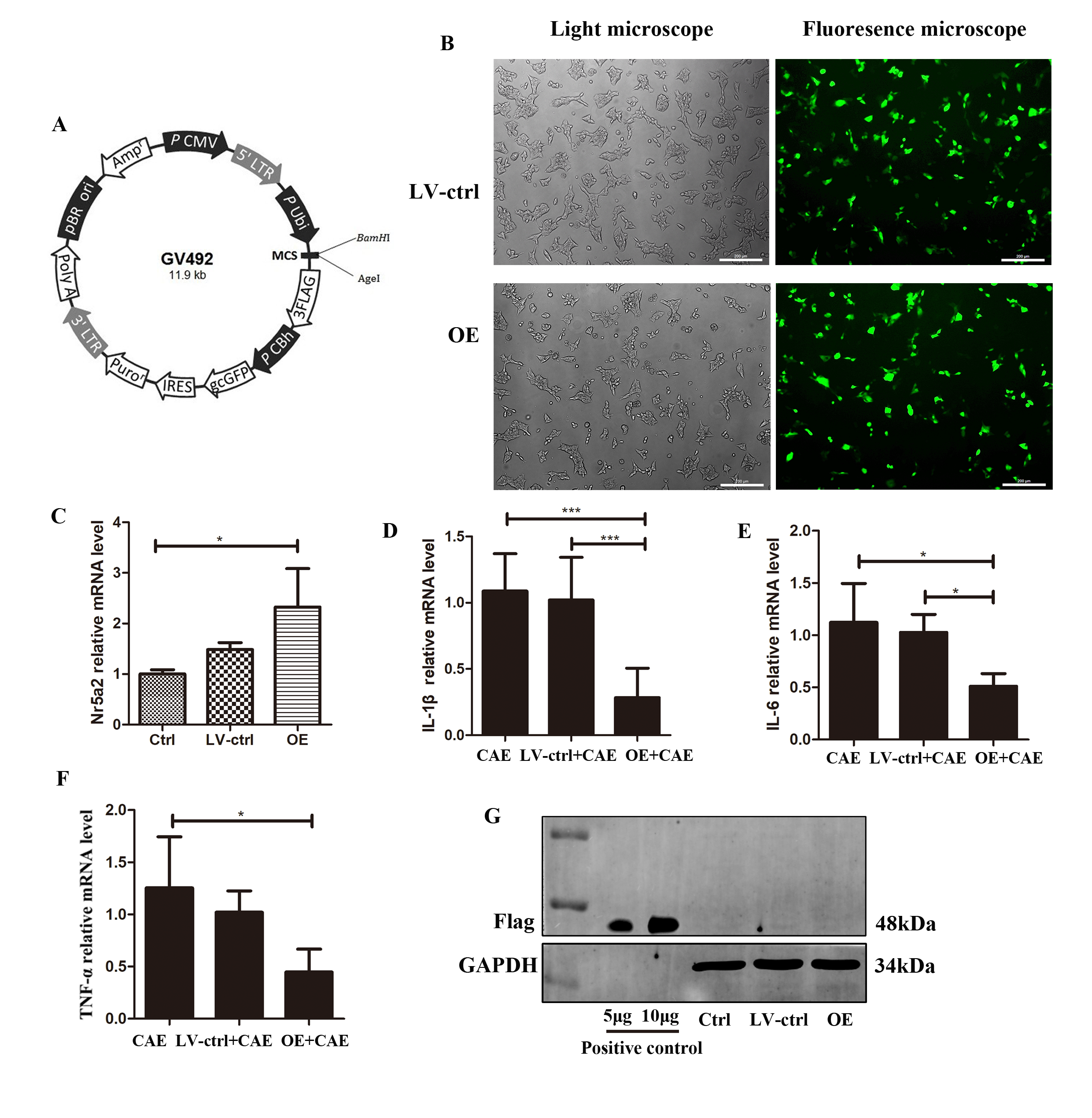

Supplement: FIGURE S1 — Construction of the lentiviral vector for Nr5a2 overexpression. (A) The component order in the lentiviral vector for Nr5a2 overexpression. (B) The infection rate of control lentiviral vector or the Nr5a2-overexpression vector in AR42J cells was assessed using a fluorescence microscope at 48 h. (C) Nr5a2 mRNA level after infection with lentiviral vector was measured by qRT-PCR at 5 days. (D–F) The effects of Nr5a2 overexpression at the transcriptional level on IL-1β, IL-6, and TNF-α mRNA levels were measured by qRT-PCR. (G) Flag protein expression was determined by Western blot after infection with lentiviral vector for 6 days. Positive control: positive control expressing the flag protein, LV-ctrl: infection with the control lentiviral vector, OE: infection with lentiviral vector overexpressing Nr5a2. Data are shown as mean ± SEM, n > 3 in each test, ∗p < 0.05, ∗∗∗p < 0.001. [file Image_1.TIF]

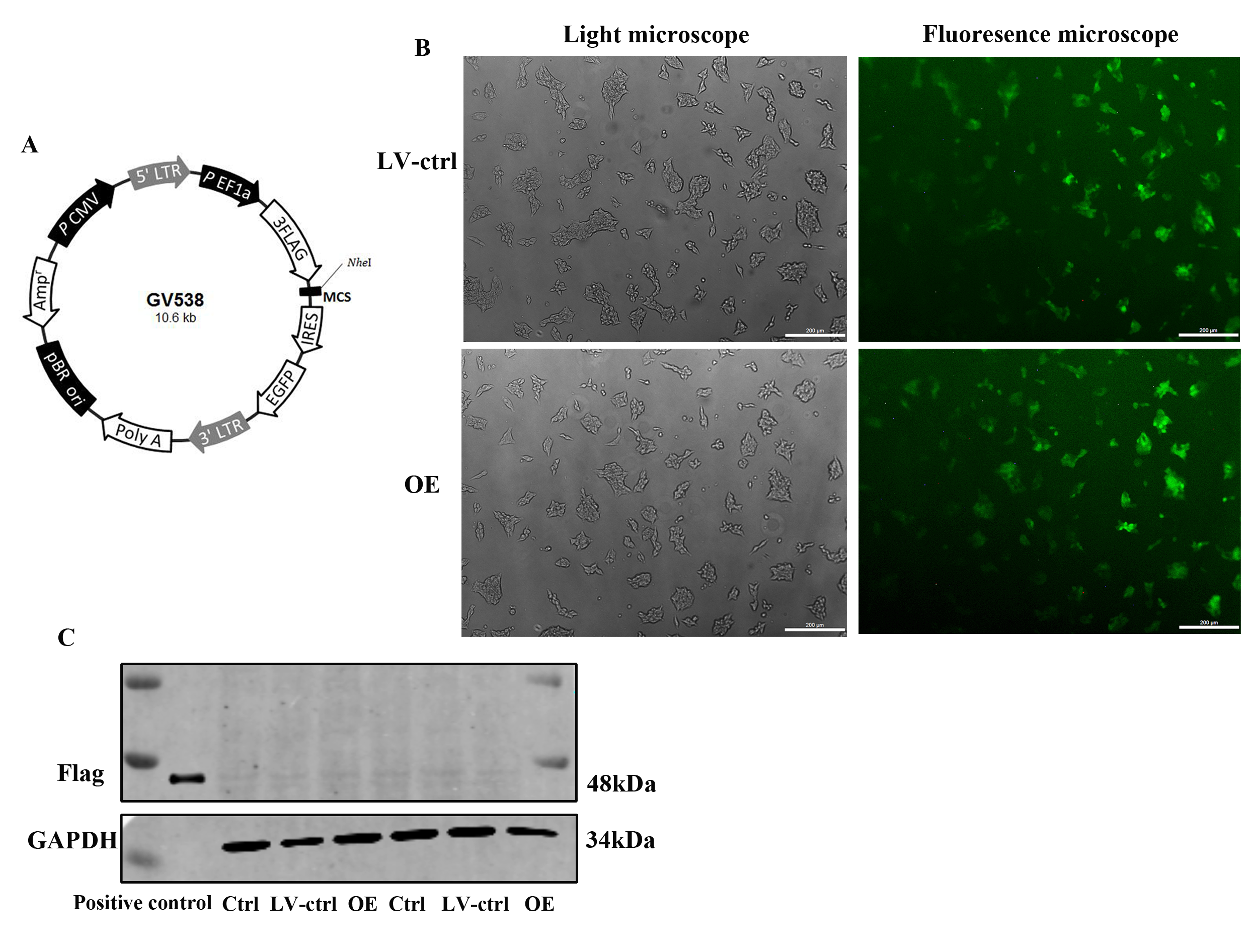

Supplement: FIGURE S2 — Re-construction of the lentiviral vector for Nr5a2 overexpression. (A) The component order in the lentiviral vector for Nr5a2 overexpression. (B) The infection rate of control lentiviral vector or the Nr5a2-overexpression vector in AR42J cells was assessed using a fluorescence microscope at 48 h. (C) Flag protein expression was determined by Western blot after infection with the lentiviral vector for 6 days. Data are shown as mean ± SEM, n > 3 in each test. [file Image_2.TIF]
